# Factors associated with one-year mortality after hospital discharge: A multicenter prospective cohort study

**DOI:** 10.1371/journal.pone.0288842

**Published:** 2023-08-09

**Authors:** Fabian D. Liechti, Lukas Bütikofer, Marco Mancinetti, Joerg D. Leuppi, Daniel Genné, Gregor John, Jacques D. Donzé

**Affiliations:** 1 Department of General Internal Medicine, Inselspital, Bern University Hospital, University of Bern, Bern, Switzerland; 2 Clinical Trials Unit, University of Bern, Bern, Switzerland; 3 Department of Medicine, Hôpital Cantonal de Fribourg, Fribourg, Switzerland; 4 Medical Education Unit, University of Fribourg, Fribourg, Switzerland; 5 Faculty of Medicine, University of Basel, Basel, Switzerland; 6 Kantonsspital Baselland, University Clinic of Medicine, Liestal, Switzerland; 7 Department of Internal Medicine, Biel Hospital Centre, Biel, Switzerland; 8 Faculty of Medicine, University of Geneva, Geneva, Switzerland; 9 Department of Internal Medicine, Geneva University Hospitals and University of Geneva, Switzerland; 10 Department of Medicine, Neuchâtel Hospital Network, Neuchâtel, Switzerland; 11 Division of General Medicine, Brigham and Women’s Hospital, Harvard Medical School, Boston, Massachusetts, United States of America; 12 Division of Internal Medicine, Lausanne University Hospital, Lausanne, Switzerland; Istanbul University-Cerrahpasa, Cerrahpasa Medical Faculty, TURKEY

## Abstract

**Objectives:**

1) To identify predictors of one-year mortality in hospitalized medical patients using factors available during their hospital stay. 2) To evaluate whether healthcare system use within 30 days of hospital discharge is associated with one-year mortality.

**Study design and setting:**

This prospective, observational study included adult patients from four mid-sized hospital general internal medicine units. During index hospitalization, we retrieved patient characteristics, including demographic and socioeconomic indicators, diagnoses, and early simplified HOSPITAL scores from electronic health records and patient interviews. Data on healthcare system use was collected using telephone interviews 30 days after discharge. Survival status at one year was collected by telephone and from health records. We used a univariable analysis including variables available from the hospitalization and 30-day post-discharge periods. We then performed multivariable analyses with one model using index hospitalization data and one using 30-day post-discharge data.

**Results:**

Of 934 patients, 123 (13.2%; 95% CI 11.0–15.4%) were readmitted or died within 30 days. Of 814 patients whose primary outcome was available, 108 died (13.3%) within one year. Using factors obtained during hospitalization, the early simplified HOSPITAL score (OR 1.50; 95% CI 1.31–1.71; *P* < 0.001) and not living at home (OR 4.0; 95% CI 1.8–8.3; *P* < 0.001) were predictors of one-year mortality. Using 30-day post-discharge predictors, hospital readmission was significantly associated with one-year mortality (OR 4.81; 95% CI 2.77–8.33; *P* < 0.001).

**Significance:**

Factors predicting one-year mortality were a high early simplified HOSPITAL score, not living at home, and a 30-day unplanned readmission.

## Introduction

Hospital readmission is usually evaluated together with death—the most severe post-discharge outcome. Post-discharge mortality can be higher in specific settings and is mainly assessed at 30 days after hospital discharge [1–3]. Few studies have investigated mortality risk at 6–12 months after hospital discharge, and most studies to date have focused on specific patient groups, *e*.*g*., geriatric patients. Among geriatric patients, malnutrition, falls, delirium, impairment in the activities of daily living, and functional impairment at the time of discharge have all been associated with death [4–8]. However, risk factors may vary according to the population studied [5, 9–11] and are challenging to identify [12].

Simple tools to help clinicians predict one-year mortality are lacking. The Hospital-patient One-year Mortality Risk (HOMR) model uses twelve predictors available during hospitalization and is the only prediction model to have been externally validated using a large population [13, 14]. However, the HOMR score is mainly based on administrative data, covers patients from all departments, and includes in-hospital mortality. It does not consider the discharge process and post-discharge care. The simplified HOSPITAL score is a validated prediction model for 30-day hospital readmission whose variables include hemoglobin and sodium levels at discharge, cancer diagnosis or discharge from an oncology unit, index type of admission (emergent *vs*. elective), number of admissions in the previous 12 months, and length of stay [15, 16]. Whether the simplified HOSPITAL score is a predictor of one-year post-discharge mortality remains unknown. Hence, little is known about the factors associated with the one-year mortality of general medical patients after hospital discharge. Whether healthcare system use after discharge is associated with mortality risk is also unknown. Our study aimed to identify factors obtained during the hospital stay or in the 30 days after hospital discharge which are associated with the all-cause mortality of general-internal medicine patients at one year after hospital discharge.

## Methods

### Study design and participants

We performed a three-month (August to October 2017) prospective, observational, multicenter cohort study. We screened all the adult patients in the general internal medicine units of four mid-sized hospitals in Switzerland whose length of stay was more than one day and who were planned to be discharged home (or to a nursing home) within the next 36 hours. Patients were screened during index hospitalization by a study nurse. Before study inclusion, all participants signed an informed consent form after receiving oral and written information. Participating patients were not reassessed if readmitted to hospital. Patients who could not provide follow-up information by telephone interview or who were unwilling or unable (*e*.*g*., cognitively impaired patients) to sign the informed consent form were excluded.

### Ethics considerations

The study complied with the Declaration of Helsinki, guidelines for Good Clinical Practice, and was approved by the relevant ethics committees for the main and subsidiary study sites (Ethics Committee for the Canton of Bern, Bern, ID 2018–00084; Ethics Committee for Northwestern and Central Switzerland, Basel; Human Research Ethics Committee of the Canton of Geneva, Geneva; Human Research Ethics Committee of the Canton of Vaud, Lausanne).

### Study outcome and predictor variables

The primary outcome investigated was all-cause mortality one year after discharge from the index admission. Secondary outcomes were unplanned readmission or death within 30 days of discharge, post-discharge healthcare use (number of hospitalization days, emergency department consultations, primary care physician [PCP] consultations), patient satisfaction with care at 30 days, and outcomes within one year after the index discharge (death, the number of unplanned readmissions, and the number of unplanned hospitalization days).

Data from the index hospitalization were collected from electronic health records and included demographic characteristics, comorbidities, laboratory values, and home visits by nurses. Demographic data and other patient characteristics were collected at baseline by interview after patient inclusion (age, sex, health insurance coverage [standard or private], place of residence [home *vs*. nursing home, urban *vs*. rural], number of people living in the same residence, PCP availability, home care [nurse visits, help in the activities of daily living], diagnoses at index admission, hospital site, and discharge destination). Based on this data we calculated the simplified HOSPITAL score which has been originally developed to predict 30-day readmission [16]. For practical reasons, i.e., to have the data available before hospital discharge, we decided to use laboratory values at admission (first available) instead of at discharge (last available) and called it early simplified HOSPITAL score (Table 1 in S1 File), which is valid since we were evaluating a different outcome and not developing a prediction model.

Data at 30 days after discharge were collected by telephone interview and included the number of emergency department consultations, PCP consultations, and unplanned readmissions. Unplanned readmission was defined as a non-elective hospitalization in any unit of any acute care hospital (*i*.*e*., not only the hospital from which the patient was originally discharged) that happened within 30 days of discharge from the index hospital. Elective hospitalization was defined as a non-urgent hospitalization scheduled at least one day before the admission day.

A trained study nurse collected information on readmission or death one year after the index admission by telephone interview with the patient (or, if unavailable, with the next of kin or a PCP). Furthermore, to improve accuracy, the study nurse consulted the participant’s medical records and their electronic health records in participating hospitals for any readmissions or death recorded. We analyzed all-cause mortality at one year after discharge from the index admission.

### Sample size, data management, and statistical analysis

This observational study was originally designed to validate the early simplified HOSPITAL score. We planned to include 600 patients in order to reach the 70 hospital readmissions, based on readmission rate of 14.5% and loss to follow-up of 20%, that would provide about 10 events per parameter in the simplified HOSPITAL score (which, as a rule of thumb, has been recommended for prediction models [16–18]). With a sample size of 70, the readmission rate could be estimated with a standard error of less than 1.5%, leading to a 95% confidence interval (95% CI) smaller than 6%.

Data management included the transfer of data from electronic health records and patient interviews to electronic Case Report Forms (eCRF, REDCap software, Vanderbilt University, Nashville, TN, USA [19]). The study database was exported using an automated procedure via the REDCap application programming interface).

All statistical analyses were done using Stata (StataCorp. Stata Statistical Software: Release 15. StataCorp LP, College Station, TX, USA, 2017) and R software (Core Team. R: A Language and Environment for Statistical Computing. R Foundation for Statistical Computing, Vienna, Austria, 2016). Categorical variables are presented using absolute and relative frequencies, and continuous variables are presented using median and interquartile range (IQR). For unplanned readmissions and death, or death alone, we calculated proportions using 95% Wilson-score confidence intervals (CIs) and cumulative incidences using the Kaplan–Meier estimator with pointwise Greenwood standard errors to construct Wald-type 95% CIs. We calculated cumulative incidences of readmission alone, with death (without readmission) as a competing event, using 95% CIs, as suggested by Choudhury [20]. Count outcomes are reported as incidence rates with 95% Poisson CIs using the observation time as offset.

Logistic regressions were used to evaluate baseline characteristics for their prognostic effect on the primary outcome. Effects are reported as an odds ratio (OR) with a 95% CI. We used complete cases (see below), and the number of observations included in each model depended on the predictor variable. Count predictor variables (number of unplanned hospitalization days within 30 days, emergency department consultations within 30 days, and PCP consultations within 30 days) were dichotomized (none *vs*. any unplanned readmissions, none *vs*. any emergency department consultations, and none *vs*. any PCP consultations, irrespective of the number). All predictors were first analyzed using a univariate logistic regression. Two multivariable models were constructed based on clinical and statistical reasoning, one containing the predictors from hospitalization and one containing the predictors available at 30 days after discharge. In the first multivariable model, all the discharge variables with a *P* < 0.2 in the univariable model were included, except for the albumin level (which was frequently missing) and comorbidities (which were rare, to avoid overfitting), *i*.*e*., age, living at home, nurse visits at home, sodium level at admission, hemoglobin level at admission, early HOSPITAL score (with laboratory values at admission), sex, insurance category, and travel time to a PCP. As an alternative analysis, we also included comorbidities (see Table 5 in S1 File). For the second multivariable model, all 30-day variables with a *P* < 0.2 were included, except albumin, *i*.*e*., any unplanned readmissions within 30 days, any emergency department consultations within 30 days, and any PCP consultations within 30 days.

### Missing data

For the 30-day outcomes, we used multiple imputations to account for missing data, using chained equations with predictive mean matching for continuous variables, logistic regression for binary variables, and multinomial logistic regressions for categorical variables. We included baseline variables that were not highly correlated or unequally distributed and imputed the 30-day outcome in the first step and all other outcome variables in the second step. A total of 20 multiple imputations were calculated and analyzed using Rubin’s rule. As a sensitivity analysis, we used observed data alone (complete cases), ignoring patients without a 30-day outcome for binary and count outcomes, and censoring them after a short observation time (1 hour) for survival outcomes. The survival time of a single patient with a readmission at an unknown date was imputed using the mean.

For one-year outcomes, only patients with a non-missing observation at follow-up were considered for the proportion of deaths, whereas the last known value was used for the number of readmissions and hospitalization days and observation times were adjusted accordingly. Cumulative incidences were calculated by censoring dropouts at the time of drop-out.

## Results

**Fig 1:**
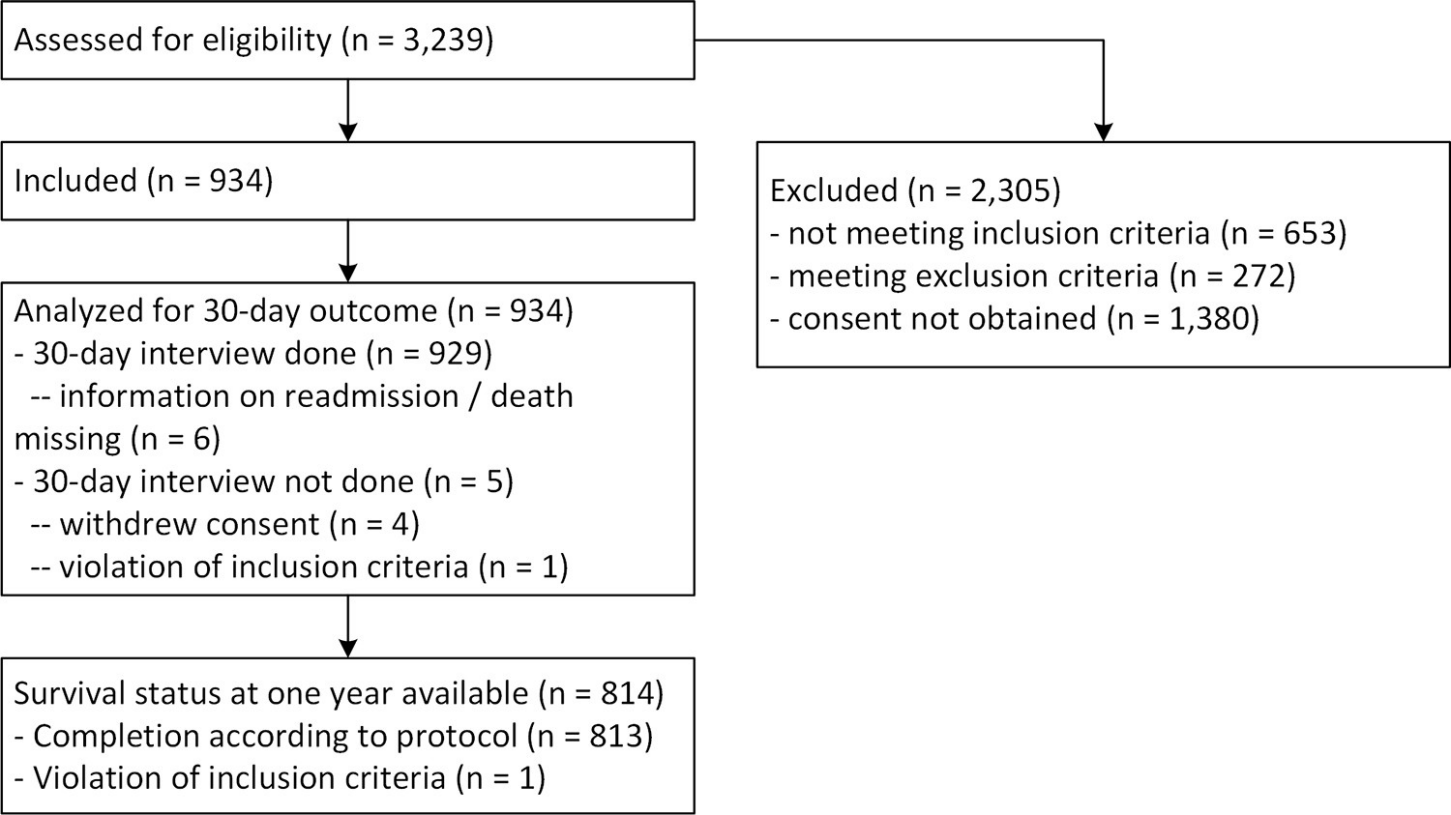
Flow chart.

Of the initial 3,239 patients screened for eligibility, 934 were included in the study (Fig 1).

Of the 934 patients included, 929 underwent an interview at 30 days, of whom the outcome of death or readmission remained unknown for 6. We therefore used multiple imputations in 11 cases for the 30-day outcome. From the 934 participants originally included, 734 interviews at one year after discharge could be done (86 died within one year, but we could not perform an interview with a next of kin or PCP, 32 withdrew consent, 79 had an incomplete follow-up, 2 were lost to follow-up, and 1 violated inclusion criteria). We were able to assess the survival status of 814 participants. Patients’ baseline characteristics are presented in Table 1. Of 934 patients, 123 (13.2%; 95% CI 11.0–15.4%) were readmitted or died within 30 days, of whom 113 (12.1%; 95% CI 10.0–14.3%) were readmitted for a total of 923 hospitalization days (incidence rate per 30 days 1.00; 95% CI 0.93–1.07). Twenty-one patients (2.2%; 95% CI 1.3–3.2%) died within 30 days. Furthermore, 102 emergency department consultations (30-day incidence rate 0.11; 95% CI 0.09–0.13) and 1,555 PCP consultations (30-day incidence rate 1.69; 95% CI 1.60–1.77) were registered within 30 days. A sensitivity analysis using complete cases showed similar results (see Table 4 in S1 File).

**Table 1:** Characteristics and demographics at admission (n = 934).

	Median [IQR] or n (%)
**Age**	71 [58, 80]
**Male sex**	526 (56)
**Health insurance**	
Standard	436 (47)
Standard and complementary	267 (29)
Semi-private	166 (18)
Private	63 (7)
No insurance	1 (0)
**Patient has a primary care physician**	919 (98)
**Travel time to primary care physician in minutes**	10 [10, 15]
**Living status**	
With spouse/partner	565 (60)
With another person	55 (6)
Alone	307 (33)
Unknown	7 (1)
**Residence type**	
Home	882 (94)
Protected housing	11 (1)
Nursing home	35 (4)
Unknown/other	6 (1)
**Home visits by a nurse**	232 (25)
**Support at home**	
for cleaning	260 (28)
for buying groceries	94 (10)
for eating	83 (9)
**Source of income**	
Employed	170 (18)
Self-employed	41 (4)
Unemployed	16 (2)
Retired	627 (67)
Invalidity insurance	35 (4)
Social security benefits	21 (2)
Unknown/other	24 (3)
**Living in an urban area**	824 (88)
**Length of hospital stay [days]**	6 [4, 9]
**Hemoglobin level at admission < 120 g/l**	257 (28)
**Diagnosis of active cancer**	180 (19)
**Sodium level at admission < 135 mmol/l**	234 (25)
**Non-elective admission**	859 (92)
**Number of hospitalizations at the same hospital in the last 12 months**	
0–1	779 (83)
2–5	150 (16)
> 5	5 (1)
**Early simplified HOSPITAL score^1^ [score points]**	3.00 [1.00, 4.00]

IQR, interquartile range. ^1^ Simplified HOSPITAL score using laboratory values at admission.

**Fig 2:**
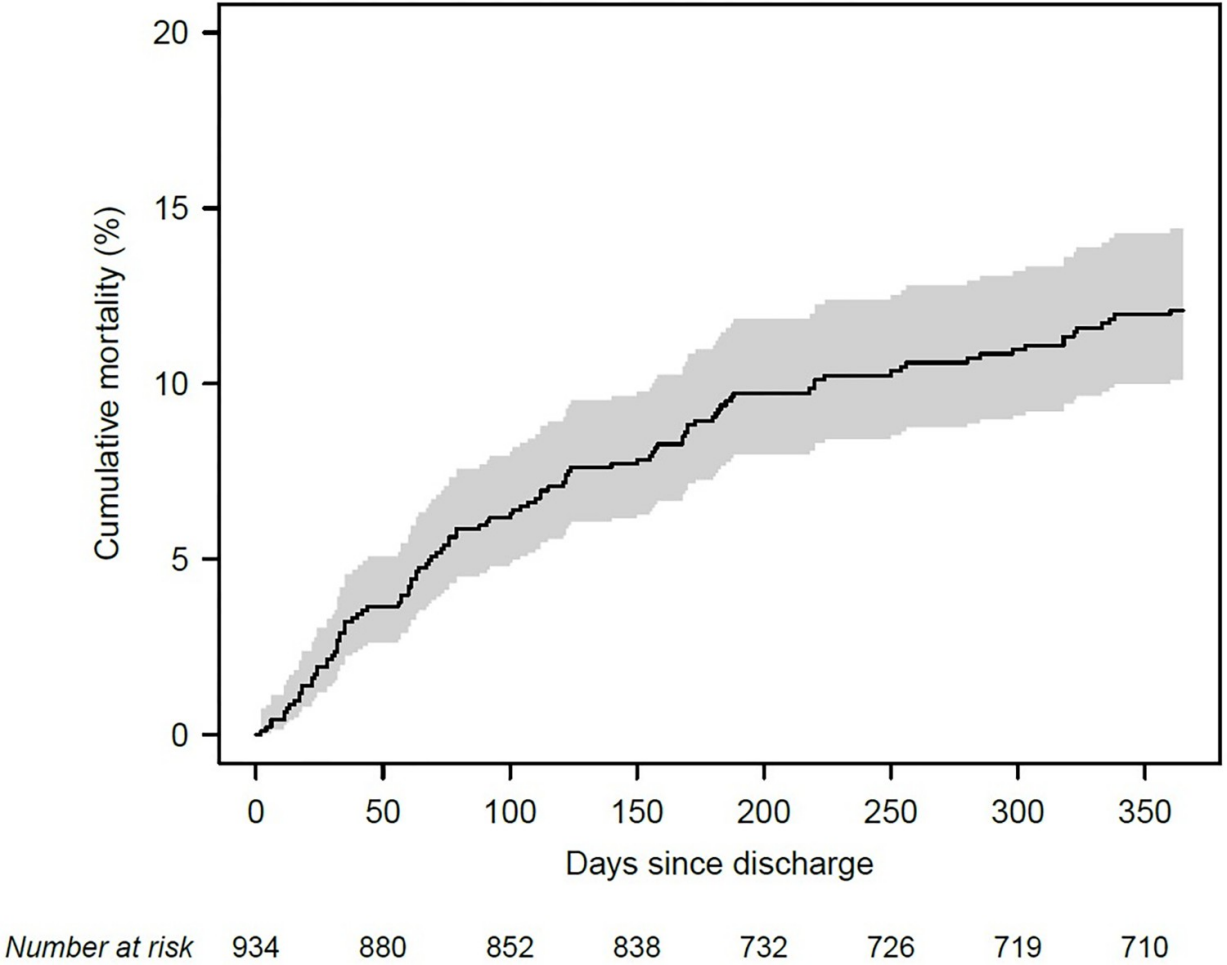
Cumulative mortality. Cumulative mortality up to one-year post-discharge with 95% confidence intervals (grey shading). One patient with missing outcome data in each of their follow-ups was censored after 1 hour.

Over the one-year follow-up period, 108 of the 814 patients whose survival status was known had died (13.3%; 95% CI 11.1–15.8%), resulting in a cumulative incidence of death of 12.1% (10.1–14.4%) at one year (Fig 2). In the same period, 689 readmissions were registered in 781 person-years (one-year incidence rate 0.84; 95% CI 0.78–0.91), corresponding to 5,717 unplanned hospitalization days.

The factors available at the time of hospital discharge that were significantly associated with increased one-year mortality in the univariable analysis (with *P* < 0.05) were older age, not living at home, home visits by nurses, dementia, active cancer, liver cirrhosis, hyponatremia, lower hemoglobin and albumin levels than at admission, and higher scores in the early simplified HOSPITAL score with laboratory results at admission (Table 2). The factors available at 30-days after discharge that were associated with increased one-year mortality were any unplanned hospitalization (OR 5.84; 95% CI 3.65–9.36; *P* < 0.001) and any emergency department consultation (OR 2.94; 95% CI 1.70–5.11; *P* < 0.001). A PCP consultation within 30 days of discharge showed a statistically non-significant negative association with one-year mortality (OR 0.63; 95% CI 0.38–1.03; *P* = 0.07).

**Table 2:** Univariable logistic regression for death at one-year—factors available during hospitalization.

	Number of observations	Odds ratio (95% CI)	*P* value
**Age [decades]**	814	1.32 (1.14–1.54)	< 0.001
**Female sex**	814	0.98 (0.65–1.48)	0.94
**Living at home**	812	0.18 (0.10–0.35)	< 0.001
**Semi-private or private insurance**	813	0.85 (0.53–1.38)	0.52
**Visits at home by a nurse**	812	3.10 (2.04–4.71)	< 0.001
**Comorbidities**			
Chronic heart failure	814	0.97 (0.55–1.72)	0.93
Coronary disease	814	1.06 (0.66–1.69)	0.81
Atrial fibrillation	814	1.30 (0.80–2.13)	0.29
Peripheral artery disease	814	1.76 (0.94–3.29)	0.08
Diabetes	814	1.12 (0.69–1.80)	0.65
Dementia	814	3.12 (1.38–7.04)	0.006
COPD	814	1.53 (0.85–2.75)	0.15
Active cancer	814	9.23 (5.88–14.50)	< 0.001
Chronic renal failure	814	1.31 (0.82–2.11)	0.26
Liver cirrhosis	814	2.61 (1.13–6.05)	0.025
Drug or alcohol abuse	814	0.82 (0.41–1.64)	0.58
Any treated psychiatric disease	814	0.58 (0.27–1.24)	0.16
**Travel time to primary care physician [min]**	797	0.99 (0.97–1.02)	0.54
**Sodium level^1^ [mmol/L]**	786	0.96 (0.92–1.00)	0.036
**Hemoglobin level^1^ [g/L]**	790	0.98 (0.97–0.98)	< 0.001
**Albumin level^1^ [g/L]**	606	0.91 (0.87–0.95)	< 0.001
**Early simplified HOSPITAL score^1^ [score points]**	814	1.58 (1.42–1.76)	< 0.001

^*1*^ Simplified HOSPITAL score using laboratory values at admission.

In our multivariable analysis using the factors available at discharge, higher scores in the early simplified HOSPITAL score (using laboratory values at admission) and not living at home were predictors of increased one-year mortality (Table 3). When we included comorbidities in the model, dementia, active cancer, and liver cirrhosis were revealed as potential risk factors for one-year mortality, and the effect of the early simplified HOSPITAL score partially disappeared as active cancer became part of the score (Table 3 in S1 File).

**Table 3:** Multivariate logistic regression for death at 1 year with predictors available during the hospital stay (N = 758).

	Odds ratio (95% CI)	*P* value
**Age [decades]**	1.17 (0.97–1.41)	0.09
**Female sex**	0.82 (0.51–1.32)	0.42
**Living at home**	0.25 (0.12–0.54)	< 0.001
**Semi-private or private insurance**	0.94 (0.54–1.66)	0.84
**Home visits by a nurse**	1.62 (0.98–2.69)	0.06
**Travel time to primary care physician**	1.00 (0.97–1.03)	0.96
**Sodium level^1^**	0.99 (0.95–1.04)	0.72
**Hemoglobin level^1^**	0.99 (0.98–1.00)	0.22
**Early simplified HOSPITAL score^1^**	1.50 (1.31–1.71)	< 0.001

^1^ Simplified HOSPITAL score using laboratory values at admission.

In our multivariable logistic regression using predictors of healthcare system use within 30 days of discharge (Table 4), a 30-day unplanned readmission was significantly associated with one-year mortality (OR 4.81; 95% CI 2.77–8.33; *P* < 0.001). Emergency department consultations were not associated with one-year mortality (OR 1.41; 95% CI 0.72–2.74; *P* = 0.32). We did not find any evidence of an association between a PCP visit within 30 days and the primary outcome (OR 0.67; 95% CI 0.40–1.13; *P* = 0.13).

**Table 4:** Multivariate logistic regression for death at 1 year with predictors available at 30 days after discharge (N = 795).

	Odds ratio (95% CI)	*P* value
**Any unplanned readmission**	4.81 (2.77–8.33)	< 0.001
**Any emergency department consultation**	1.41 (0.72–2.74)	0.32
**Any primary care provider consultations**	0.67 (0.40–1.13)	0.13

## Discussion

This prospective, observational, multicenter cohort study included a mixed population of general internal medicine inpatients planned to be discharged home, and aimed at evaluating potential predictors of the 13% one-year mortality reported in our four participating hospitals. The early simplified HOSPITAL score and not living at home before hospitalization were used as independent predictors in our model using data collected during hospitalization. Unplanned readmissions were associated with higher one-year mortality in the model using 30-day post-discharge data. Factors associated with increased one-year mortality in the univariable model were older age, not living at home, home visits by nurses, dementia, active cancer, laboratory values at admission (hyponatremia, low hemoglobin or albumin), the early simplified HOSPITAL score, and any emergency department consultations within 30 days of discharge.

Identifying risk factors assessed during patients’ hospital stays or in their 30-day post-discharge period—as described in the present study—could help improve the transition of care, including discharge planning [21, 22]. Recognizing patients at risk of an unfavorable outcome is an important prerequisite for distributing resources and targeting interventions such as multimodal geriatric assessment (which has been shown to reduce six-month post-discharge mortality) to the patients likely to benefit most from it [23, 24]. The association between mortality and the early simplified HOSPITAL score has several potential explanations. First, the HOSPITAL score was developed to predict unplanned readmissions, which, in turn, have been associated with mortality at 30 days [25] and one year [26]. Second, many items in the score have been recognized as being linked to “post-hospitalization stress” [15, 27]. Third, early readmission and post-discharge mortality are probably dependent on similar socioeconomic factors, e.g., financial incentives for hospitals intended to decrease readmission rates might result in increased mortality rates or organizational structures in hospitals such as resident’s workloads influence patient outcomes [2, 3, 28, 29]. Finally, when comorbidities are added to the model, the effects of the early simplified HOSPITAL score disappear, suggesting that it contains similar information to the comorbidities.

The importance of timely follow-up consultations with a PCP after hospital discharge, as an aid to avoiding readmissions, has been shown earlier [30]. Indeed, in the present study, the varied patterns of healthcare use within 30 days of discharge were associated with alterations in one-year mortality, *i*.*e*., mortality was higher among those with unplanned readmissions. However, we did not find an association with PCP consultations. These findings add to the observation made in the development of the HOMR model, where hospitalizations in the year before index hospitalization appeared to be an independent predictor of one-year mortality [13].

Our study, nonetheless, had some limitations. First, the study centers were all in Switzerland, meaning our findings may not be generalizable to other healthcare settings or locations. However, the study did include a diverse range of patients admitted to hospitals of varying sizes in different regions of the country. Second, we only included predictors easily obtainable from commonly available electronic health records. We cannot exclude the presence of other risk factors, including patients’ functional status, which can be difficult to assess due to the acute pathologies leading to hospital admission. Third, the primary outcome data from the one-year follow-up of 120 patients were missing from the analysis, corresponding to a follow-up at one year after discharge of 87% when compared to the data available from 30-day outcomes. We decided to include all the available data for 30-day outcomes because those with missing outcomes after one year might differ from those with available information, and thus they might provide important information regarding the endpoint of early post-discharge mortality and re-hospitalization within 30 days of discharge. Moreover, the number of events (108 deaths) was still large enough to include up to 10 parameters in our multivariable regression model [17, 18].

In conclusion, higher scores in the early simplified HOSPITAL score and not living at home were predictors of one-year post-discharge mortality in the model using data available at hospital discharge, and readmission within 30 days of hospital discharge was a predictor in the model based on healthcare system use post-discharge. The latter factor could aid treating physicians in their decision-making and provide options for interventions aimed at improving the transition of care and reducing post-discharge mortality.

## Supporting information

S1 File (DOCX)
