# Supplementary material for: Factors associated with one-year mortality after hospital discharge: A multicenter prospective cohort study
Source: PLoS One. 2023 Aug 9;18(8):e0288842. doi: 10.1371/journal.pone.0288842 (PMC10411790; doi:10.1371/journal.pone.0288842)
Supplement: S1 File — (DOCX) [file pone.0288842.s001.docx]

## Supplementary Materials

Table 1. Calculation of the early simplified HOSPITAL score is based on calculation of the simplified HOSPITAL score but uses laboratory values available at admission instead of at discharge. 0 to 4 score points indicate unlikely 30-day readmission, ≥ 5 score points indicate likely 30-day readmission.

| **Variable** | **Early simplified HOSPITAL score (number of points)** |
| --- | --- |
| Hemoglobin level at admission < 120 g/l | 1 |
| Cancer diagnosis or discharge from an oncology division | 2 |
| Sodium level at admission y 135 mmol/l | 1 |
| Index type of admission: non-elective | 1 |
| Number of hospital admissions during the previous 12 months |  |
| 0 to 1 | 0 |
| 2 to 5 | 2 |
| ≥ 5 | 5 |
| Length of stay ≥ 5 days | 2 |
| **Total** | **12** |

Table 2. Baseline characteristics.

| **Comorbidities*** | **Total (N = 934), n (%)** |
| --- | --- |
| Chronic heart failure | 134 (14%) |
| Coronary disease | 247 (26%) |
| Atrial fibrillation | 170 (18%) |
| Peripheral artery disease | 86 (9.2%) |
| Diabetes | 214 (23%) |
| Dementia | 30 (3.2%) |
| COPD | 94 (10%) |
| Active cancer | 138 (15%) |
| Chronic renal failure | 196 (21%) |
| Liver cirrhosis | 30 (3.2%) |
| Drug or Alcohol Abuse | 96 (10%) |
| Any treated psychiatric disease | 98 (10%) |
| * Does not sum up as multiple entries are possible. | |

Table 3. 30-day outcomes, based on all patients and using multiple imputations (N = 934). n = number of events; N = number of patients; CI = confidence interval; * = cumulative incidence with death (without readmission) as a competing event.

|  | **n/N** | **Proportion in % (95% CI)** |
| --- | --- | --- |
| Unplanned readmission or death within 30 days | 123/934 | 13.2 (11.0–15.4) |
| Death within 30 days | 21/934 | 2.2 (1.3–3.2) |
| Unplanned readmission within 30 days | 113/934 | 12.1 (10.1–14.3)* |
| High satisfaction with quality of transition of care between hospital and home | 741/934 | 79.3 (76.6–82.0) |
|  | **n/person-time (days)** | **Incidence rate per 30 days (95% CI)** |
| Unplanned hospitalization days within 30 days | 923/27,664 | 1.00 (0.93–1.07) |
| Emergency department consultations within 30 days | 102/27,664 | 0.11 (0.09–0.13) |
| Primary care provider consultations within 30 days | 1555/27,664 | 1.69 (1.60–1.77) |

Table 4. 30-day outcomes based on complete cases for each outcome. n = number of events; N = number of patients; CI = confidence interval; * = cumulative incidence with death (without readmission) as a competing event.

|  | **n/N** | **Proportion in % (95% CI)** |
| --- | --- | --- |
| Unplanned readmission or death within 30 days | 122/924 | 13.2 (11.2–15.5) |
| Death within 30 days | 21/926 | 2.3 (1.5–3.4) |
| Unplanned readmission within 30 days | 111/925 | 12.0 (10.0–14.2)* |
| High satisfaction with quality of transition of care between hospital and home | 701/883 | 79.4 (76.6–81.9) |
|  | **n/person-time (days)** | **Incidence rate per 30 days (95% CI)** |
| Unplanned hospitalization days | 900/27,394 | 0.99 (0.92–1.05) |
| Emergency department consultations | 100/27,394 | 0.11 (0.09–0.13) |
| Primary care provider consultations | 1,498/26,759 | 1.68 (1.60–1.77) |

Table 5. Multivariate logistic regression for death at one year, with predictors available at discharge, including comorbidities (N = 758).

|  | **Odds ratio (95% CI)** | **P-value** |
| --- | --- | --- |
| Age [decades] | 1.21 (0.98–1.50) | 0.08 |
| Female sex | 0.97 (0.58–1.62) | 0.91 |
| Living at home | 0.16 (0.07–0.37) | < 0.001 |
| Semi-private or private insurance | 0.95 (0.51–1.75) | 0.87 |
| Home visits by a nurse | 1.71 (0.99–2.96) | 0.06 |
| Travel time to primary care physician [min] | 1.00 (0.97–1.03) | 0.85 |
| Sodium level at admission [mmol/L] | 0.99 (0.95–1.04) | 0.78 |
| Hemoglobin level at admission [g/L] | 0.99 (0.98–1.00) | 0.23 |
| Simplified HOSPITAL score with laboratory results at admission | 1.13 (0.95–1.34) | 0.16 |
| Peripheral artery disease | 1.54 (0.70–3.38) | 0.28 |
| Dementia | 3.20 (1.22–8.40) | 0.018 |
| COPD | 1.04 (0.49–2.19) | 0.92 |
| Active cancer | 9.27 (4.75–18.07) | < 0.001 |
| Liver cirrhosis | 4.12 (1.48–11.49) | 0.007 |
| Any treated psychiatric disease | 0.43 (0.17–1.08) | 0.07 |
